# Need for improved regulation of tobacco e-commerce

**DOI:** 10.1136/tc-2023-058515

**Published:** 2024-01-08

**Authors:** Eric C Leas

**Affiliations:** 1 Herbert Wertheim School of Public Health and Human Longevity Science, University of California, San Diego, La Jolla, CA, USA

**Keywords:** Media, Public policy, Surveillance and monitoring, Tobacco industry

 In 1995, Mark Hayman^1^ first introduced *Tobacco Control* to e-commerce when he described what was then only a handful of websites that essentially functioned as mail-order catalogues with pictures and descriptions of tobacco products that could be ordered by calling a landline. Today, tobacco can be ordered from websites within seconds on any computer, smartphone or tablet at any time and place. E-commerce is the fastest growing segment of the tobacco marketplace, valued at US$16.7 billion in 2023 in the USA alone, and vaping e-commerce is the subsegment experiencing the largest growth.^2^

The COVID-19 pandemic was a major driver of the recent growth in e-commerce. Numerous brick-and-mortar retailers and particularly vape shops learnt to use e-commerce to avoid closing during the pandemic, when they were not considered essential businesses. Even after reopening, many brick-and-mortar retailers have continued to maintain their e-commerce platforms and operate as ‘brick-and-click’ retailers; finding that operating both platforms is more lucrative than just one and that e-commerce allows them to circumvent some policies such as flavour sales restrictions.^3^ The climate of COVID-19 also created a new demand among consumers who discovered the benefits of e-commerce, including competitive pricing, convenience and access to a wider variety of products.^4^ Survey data collected during the pandemic demonstrates that e-commerce became the primary method youth used to access vaping products, when nearly 40% of youth and young adult vapers purchased their pod-based or other refillable e-cigarettes online.^5^

This shift to e-commerce is concerning. The current e-commerce landscape has allowed tobacco retailers to operate with blatantly irresponsible marketing and sales tactics that can facilitate underage purchasing. Pearson *et al*^6^ recently identified TikTok accounts that posted videos linking to websites that sold e-cigarettes via ‘discreet shipping’—packaging and shipping that obfuscates the true contents by masquerading as cosmetics—and reassured minors that they would not be asked to present an ID or sign for a package when it was delivered. Bertrand *et al*^7^ found that 49 of 64 online vape shops they analysed allowed visitors to merely type a birthday or choose the ‘21 or older’ option to enter the site, and only 19 of 64 required an ID to be submitted during checkout procedures. A lawsuit that the state of California settled against Juul Labs also presented evidence demonstrating that Juul allowed users to repeatedly attempt purchases on their website after failing age verification. Not only that the company also sent thousands of Juul devices and pods purchased on their website to addresses with clearly fictitious customer names including ‘Beer Can’ and ‘Patricia Juul’.^8^

Jurisdictions should take action to regulate tobacco e-commerce. To begin, regulators need to unambiguously define whether tobacco e-commerce is legal in their jurisdiction. Most jurisdictions currently permit tobacco e-commerce at least in theory—ie, they have not clearly defined whether or not it is legal—but 52 countries^9^ and some local jurisdictions such as San Diego County have outright banned it. Second, if tobacco e-commerce is permitted, jurisdictions need to clarify whether e-commerce retailers are subject to tobacco retailer licensing programmes and the policies enforced through these programmes, such as taxes and flavour sales restrictions. Any retailer who sells tobacco—whether online or offline—should be recognised as a tobacco retailer, and policies should cover all retailers selling to customers within a jurisdiction, irrespective of whether the retailer is physically located in the jurisdiction or somewhere else. Finally, although it is a much more daunting task than when Mark Hayman first showcased tobacco e-commerce websites in 1995,^1^ surveillance of online retailers is sorely needed to guide enforcement. Most jurisdictions conduct routine inspections of brick-and-mortar retailers, but few if any jurisdictions routinely inspect e-commerce retailers. The need for improved regulation and monitoring of e-commerce has never been greater than it is right now.
